# Shading-Dependent Greening Process of the Leaves in the Light-Sensitive Albino Tea Plant ‘Huangjinya’: Possible Involvement of the Light-Harvesting Complex II Subunit of Photosystem II in the Phenotypic Characteristic

**DOI:** 10.3390/ijms241210314

**Published:** 2023-06-18

**Authors:** Ying-Qi Wang, Jing-Jing Ye, Hong-Zhiyuan Yang, Da Li, Xiao-Xiang Li, Yong-Kang Wang, Xin-Qiang Zheng, Jian-Hui Ye, Qing-Sheng Li, Yue-Rong Liang, Jian-Liang Lu

**Affiliations:** 1 Tea Research Institute, Zhejiang University, Hangzhou 310058, China; wang_yq@zju.edu.cn (Y.-Q.W.); 12116074@zju.edu.cn (J.-J.Y.); 22216225@zju.edu.cn (H.-Z.Y.); 22016069@zju.edu.cn (X.-X.L.); wongykang@zju.edu.cn (Y.-K.W.); xqzheng@zju.edu.cn (X.-Q.Z.); jx0515@163.com (J.-H.Y.); yrliang@zju.edu.cn (Y.-R.L.); 2 Institute of Sericulture and Tea, Zhejiang Academy of Agricultural Sciences, Hangzhou 310021, China; lida@zaas.ac.cn (D.L.); liqs@zaas.ac.cn (Q.-S.L.)

**Keywords:** *Camellia sinensis*, albino phenotype, chloroplast ultrastructure, chlorophyll-binding protein, photosystem II

## Abstract

The light-sensitive albino tea plant can produce pale-yellow shoots with high levels of amino acids which are suitable to process high-quality tea. In order to understand the mechanism of the albino phenotype formation, the changes in the physio-chemical characteristics, chloroplast ultrastructure, chlorophyll-binding proteins, and the relevant gene expression were comprehensively investigated in the leaves of the light-sensitive albino cultivar ‘Huangjinya’ (‘HJY’) during short-term shading treatment. In the content of photosynthetic pigments, the ultrastructure of the chloroplast, and parameters of the photosynthesis in the leaves of ‘HJY’ could be gradually normalized along with the extension of the shading time, resulting in the leaf color transformed from pale yellow to green. BN-PAGE and SDS-PAGE revealed that function restoration of the photosynthetic apparatus was attributed to the proper formation of the pigment-protein complexes on the thylakoid membrane that benefited from the increased levels of the LHCII subunits in the shaded leaves of ‘HJY’, indicating the low level of LHCII subunits, especially the lack of the Lhcb1 might be responsible for the albino phenotype of the ‘HJY’ under natural light condition. The deficiency of the Lhcb1 was mainly subject to the strongly suppressed expression of the *Lhcb1.x* which might be modulated by the chloroplast retrograde signaling pathway GUN1 (GENOMES UNCOUPLED 1)-PTM (PHD type transcription factor with transmembrane domains)-ABI4 (ABSCISIC ACID INSENSITIVE 4).

## 1. Introduction

The tea plant [*Camellia sinensis* (L.) O. Kuntze] is one of the most popular beverage crops worldwide [1]. During its long-term natural evolution and thousands of years of artificial selection practices, the tea plant has evolved into a variety of germplasms, including abundant mutants with albino leaves [2]. The albino tea plants have received extensive attention in research and application in recent decades since their tender shoots and leaves are rich in amino acids and can be used to make high-quality teas [3]. According to the exact leaf color and response to environmental factors, these albino tea germplasms can be roughly divided into light-sensitive and temperature-sensitive types. The tender leaves of the former will turn pale green or yellow under high light conditions and those of the latter will become white or pale green at low temperatures, respectively [4,5]. However, due to the lack of fully developed chloroplasts for photosynthesis, the leaves of the albino germplasms can only biosynthesize limited carbohydrates, tetrapyrroles, and other compounds necessary for growth and development [6]. Thus, seedling cuttings can hardly survive in the early years after transplantation. Therefore, it is very important to carry out studies on the phenotype formation mechanism of the albino germplasms since the relevant findings will help to develop novel cultivation measures for improving the survival rate after transplantation.

Cultivar ‘Huangjinya’ is a typical light-sensitive albino germplasm, and its leaves are bright yellow under natural or high light conditions but can gradually turn to normal green after being shaded or under low light conditions [7]. The abnormal phenotype of the leaves was considered to be mainly related to deficiency of the chlorophylls [8] or development blockage of the chloroplast [9], but its exact mechanism seemed to be complex and be associated with multiple regulatory pathways and metabolic processes [4,10]. When the ‘Huangjinya’ grew under natural light conditions, disrupted membrane system, dysfunctional cell compartmentalization, and decreased grana thylakoids had been observed in mesophyll cells of the albino leaves, while under shading conditions, normalized chloroplasts gradually appeared and well-stacked grana lamellae significantly increased [5]. Similar ultrastructure changes had also been observed in the leaves of another two albino cultivars ‘Baijiguan’ and ‘Zhonghuang 1#’ [9,11]. These suggested that the structure and function of the chloroplast are reversible in the leaves of light-sensitive albino tea cultivars grown at different levels of light, and defects of the chloroplast can be reversed under low light conditions. According to the iTRAQ proteomic results, the abnormal biosynthesis of the proteins involved in the development of the chloroplasts would inhibit the accumulation of the chlorophylls in the leaves of ‘Huangjinya’ [12] through down-regulating the expressions of the chlorophyll synthesis-related genes and up-regulating the chlorophyll degradation related genes [9]. Similar inhibition of the chlorophyll biosynthesis had also been witnessed in light-sensitive albino cultivars ‘Zhonghuang 2#′ [11]. In the albino leaves of the ‘Huangjinya’, the expressions of the genes encoding the photosystem II (PSII) complex subunits and light-harvesting complex (LHC) subunits were remarkably suppressed [7], which would inhibit the accumulation of the CP43 and CP47, consequently accelerate the degradation of D1 and D2 [13], and finally induce the collapse of photosynthesis. Comparison between the albino leaves and re-greened ones of the ‘Huangjinya’ showed that the differentially expressed genes (DEGs) were highly enriched in the pathways of photosynthesis and the photosynthetic antenna proteins [14]. Similar findings had also been observed in other albino cultivars, such as ‘Baijiguan’ [9].

Many attempts had been conducted and the differences in the subcellular structures, physio-chemical characteristics, and genetic traits had been obtained by comparing the albino leaves and the fully re-greened ones [10,14], but these were far from elucidating the mechanism of the albino phenotype formation in the mutant tea plant since it was difficult to effectively distinguish between genetic effects and environmental impacts for these environment- dependent albino phenotypes. In this study, the photosynthetic efficiency, photosynthetic pigments content, thylakoid architecture, the protein level of PSII and LHCII complex subunits, as well as the expression of the genes associated with photosynthesis were comprehensively monitored in the leaves of the light-sensitive albino cultivar ‘Huangjinya’ during short-term (0–144 h) shading treatment. The result showed abnormal metabolism of the photosynthetic pigments and dysfunction of chloroplast might be attributed to lack of stable assembly of the LHCII subunits, which provided a chance for insight into the formation mechanism of abnormal phenotype in light-sensitive albino tea plant.

## 2. Results

### 2.1. Changes in Phenotype Characteristics during Shading Treatment

**Figure 1:**
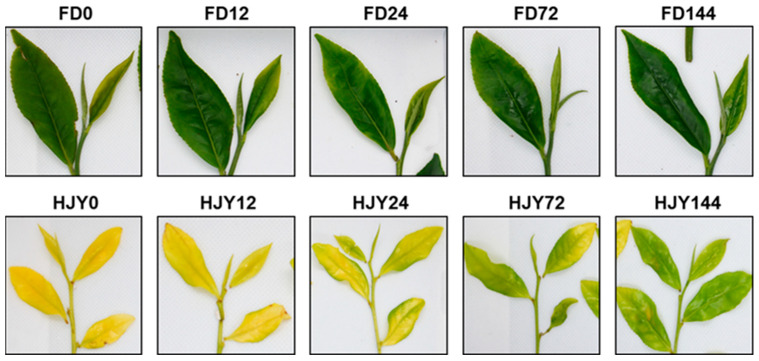
Changes in the shoot color of the ‘HJY’ and ‘FD’ during short-term shading treatment. After shading, light transmittance on the plucking table was around 10.66%. HJY–HJY144 and FD0–FD144 indicated the shoots of the ‘HJY’ and ‘FD’ after being shaded for 0–144 h.

The shoot color of the albino cultivar ‘HJY’ changed dramatically from pale yellow to light green as the shading time increased, and a significant change could be distinguished with the naked eye even after just being shaded for 24 h. In contrast, the normal green cultivar ‘FD’ exhibited a minimal alteration in the color of the shoot (Figure 1). It was clear that the albino phenotype of ‘HJY’ was mainly induced by strong light and could be reversed after being shaded.

**Figure 2:**
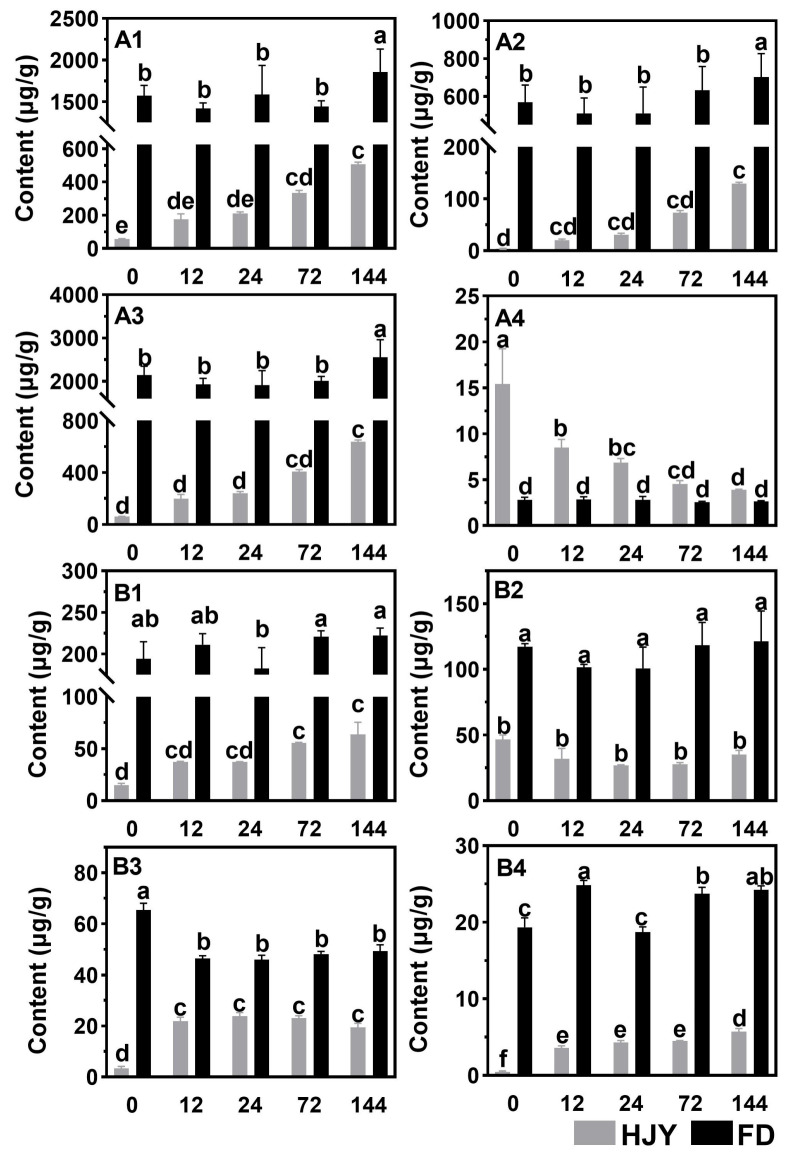
The changes in contents of photosynthetic pigments in the leaves of ‘HJY’ and ‘FD’ during short-term shading treatment (0–144 h). (**A1**), chlorophyll a; (**A2**), chlorophyll b; (**A3**), total chlorophylls; (**A4**), ratio of chlorophyll a to b; (**B1**), β-carotene; (**B2**), lutein; (**B3**), violaxanthin; (**B4**), neoxanthin. The horizontal axis represented the shading time (h). The different letters above the columns indicated a significant difference at *p* < 0.05 (one-way ANOVA, Duncan).

The contents of photosynthetic pigments were measured by HPLC, and the results were shown in Figure 2. During the shading treatment, contents of chlorophyll a and b increased slightly in the leaves of ‘FD’, but the ratio of chlorophyll a to b (2.56~2.82) hardly changed. On the contrary, the contents of chlorophyll a, b, and total chlorophylls in the leaves of ‘HJY’ increased by 7.9, 32.6, and 9.5 folds along with the extension of shading time from 0 to 144 h, although they were much lower than those in ‘FD’. The ratio of chlorophyll a to b decreased dramatically from 15.43 to 3.92 due to the unbalanced increase of their contents. Similarly, all the carotenoids in the leaves of ‘HJY’ were lower than that in the leaves of ‘FD’ with or without shading treatment. The contents of β-carotene, violaxanthin, and neoxanthin increased sharply and significantly in the leaves of ‘HJY’ after being shaded. Interestingly, the level of lutein in ‘HJY’ varied reversely with violaxanthin during the shading treatment, which was consistent with the response of the xanthophyll cycle to different light intensities. This implied that the obstacle of photosynthetic pigment synthesis and/or maintenance under natural light conditions might be one of the most fundamental reasons for the albino phenotype of ‘HJY’, although low content of photosynthetic pigments might not be the initial inducement. An increase in the content of photosynthetic pigments could reflect the functional recovery of photosynthetic apparatus after shading treatment.

**Figure 3:**
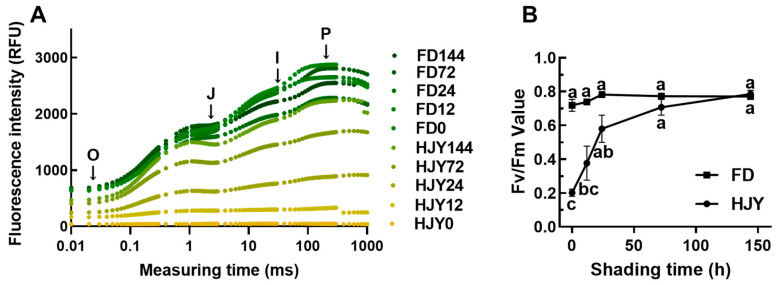
Variation of the OJIP curve (**A**) and the Fv/Fm value (**B**) in the leaves of ‘HJY’ and ‘FD’ during short-term shading treatment. O, J, I, and *p* represented the origin, junction, inflection, and maximal fluorescence intensities, respectively. The different letters indicated significant differences at *p* < 0.05 (one-way ANOVA, Duncan).

To understand the effect of the shading on the photosynthesis of the leaves, the chlorophyll a fluorescence was measured by Handy PEA. As shown in Figure 3A, the intensity of chlorophyll fluorescence was quite low in the leaves of ‘HJY’ under natural light conditions, which was greatly different from that of ‘FD’. Fluorescence intensity increased remarkably in the leaves of ‘HJY’ after being shaded for 24 h, and reached the values close to a normal OJIP curve after 144 h. Meanwhile, the OJIP curve in the leaves of ‘FD’ changed a little with an extension of the shading time. The change in fluorescence intensity in the leaves of ‘HJY’ was consistent with the observed variation in the leaf color and pigments. This suggested that the activity of PSII in the ‘HJY’ might be inhibited by strong light, and the photoinhibition could be relieved after shading treatment. 

According to the theory of energy flow in the photosynthetic membrane [15], the key biological parameter Fv/Fm was calculated (Figure 3B). In the leaves of ‘HJY’ under natural light conditions, the Fv/Fm value was significantly less than that of ‘FD’; but it enhanced sharply along with an extension of the shading time, after being shaded for more than 72 h, even reached to the value as high as ‘FD’. Interestingly, the Fv/Fm value in the leaves of ‘FD’ hardly changed after shading treatment. This implied that the photosynthetic efficiency in the leaves of ‘HJY’ would be significantly inhibited by natural light, but be markedly restored by short-term shading treatment.

### 2.2. Changes in Chloroplast Ultrastructure during Shading Treatment

**Figure 4:**
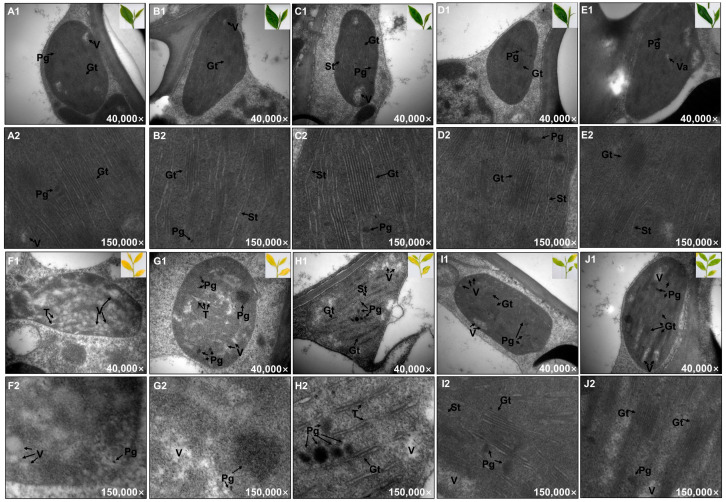
The changes in chloroplast ultrastructure during short-term shading treatment (0–144 h). (**A1**,**A2**), ‘FD’ leaves under natural light condition; (**B1**,**B2**), ‘FD’ leaves shaded for 12 h; (**C1**,**C2**), ‘FD’ leaves shaded for 24 h; (**D1**,**D2**), ‘FD’ leaves shaded for 72 h; (**E1**,**E2**), ‘FD’ leaves shaded for 144 h; (**F1**,**F2**), ‘HJY’ leaves under natural light condition; (**G1**,**G2**), ‘HJY’ leaves shaded for 12 h; (**H1**,**H2**), ‘HJY’ leaves shaded for 24 h; (**I1**,**I2**), ‘HJY’ leaves shaded for 72 h; (**J1**,**J2**), ‘HJY’ leaves shade for 144 h. T, thylakoid; Gt, grana thylakoid; St, stroma thylakoid; Pg, plastoglobule; V, void.

In order to clarify the reasons for chlorophyll fluorescence changes, the ultrastructure in the leaves of ‘HJY’ and ‘FD’ during short-term shading treatment was investigated using TEM, and the key parameters related to chloroplasts and subcellular organelles were quantified using Imaris10. Ultrastructure of the chloroplast including the thylakoid, grana thylakoid, stroma thylakoid, void, and plastoglobule, was observed in the leaves of ‘FD’ under natural light conditions, and barely changed during shading treatment (Figure 4(A1–E1,A2–E2)). On the contrary, abnormal ultrastructures were observed in the leaves of ‘HJY’ under natural light conditions (Figure 4(F1,F2)). In particular, the double-layer membrane of the chloroplast was blurred, and numerous aggregated low-electron-density voids filled in the chloroplast, but thylakoid, grana thylakoid, or stroma thylakoid could not be observed clearly. As the shading time extended, the ultrastructure of the chloroplasts gradually turned to normalness (Figure 4(G1–J1,G2–J2)) resulting in an increase in the average area of chloroplast from 3.80 μm^2^ to 4.36–5.44 μm^2^ in the leaves of ‘HJY’, accompanied by a significant decline in the number of voids and a progressive increase in the thickness, number density and area density of thylakoid. After 72 h of shading treatment, the internal structure of the chloroplasts in the leaves of ‘HJY’ was similar to that of ‘FD’ (Figure 4(D1,D2,I1,I2)). In the leaves of ‘HJY’ shaded for 144 h, nearly 30 thylakoids could be observed in each chloroplast, which was nine times more than that observed in leaves under natural light conditions (Figure 4(J1,J2)). Furthermore, thylakoids occupied approximately 43.8% of the chloroplast internal area in leaves shaded for 144 h, and the area density of thylakoid increased by nearly 46 times compared to that in the leaves of ‘HJY’ under natural light conditions (Table 1). This suggested that the restoration of both the number and area of thylakoids in the leaves of ‘HJY’ could be one of the most important events during the short-term shading treatment. The number and area density of plastoglobules in the chloroplasts of ‘HJY’ increased significantly within 24 h after shading and then slightly decreased along with a further extension of the shading time since the thylakoid membrane structure recovered. However, the area density of plastoglobules in leaves shaded for 144 h was 6 times higher than that in leaves under natural light conditions, indicating that the aggregated plastoglobules might be disassembled and participated in the reconstruction of chloroplast ultrastructure. In addition, the event of the increase in the index of the thylakoids and plastoglobules was notably earlier than that of the photosynthetic pigments and capacity.

### 2.3. Changes in the PPCs during Shading Treatment

**Figure 5:**
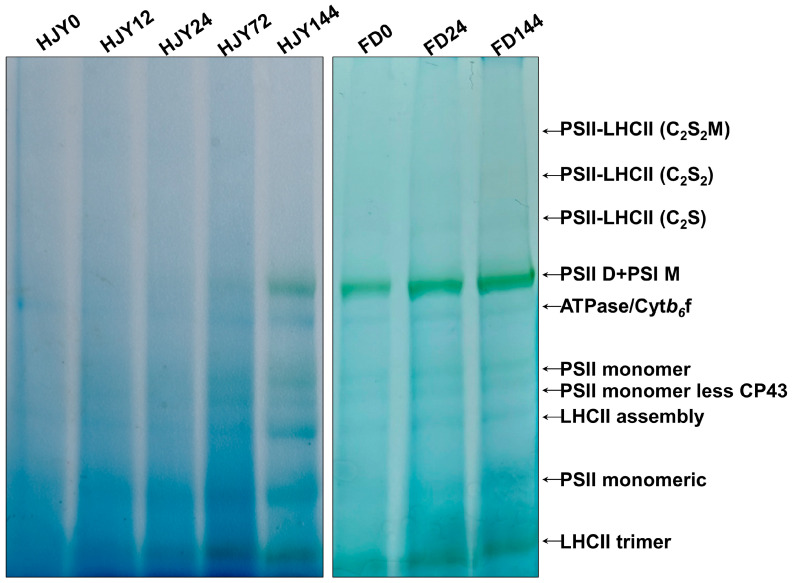
The variation of pigment-protein complexes during shading treatment. HJY0–HJY144 and FD0–FD144 indicated the leaves of ‘HJY’ and ‘FD’ shaded for 0–144 h. PSII-LHCII (C_2_S_2_M), two PSII core complexes with two stable-binding LHCII trimers, one moderate-binding LHCII trimer, and minor antenna proteins. PSII-LHCII (C_2_S_2_), two PSII core complexes with two stable-binding LHCII trimers and minor antenna proteins. PSII-LHCII (C_2_S), two PSII core complexes with one stable-binding LHCII trimer and minor antenna proteins; PSII D + PSI M, PSII dimer, and PSI monomer.

Due to the abnormalities in the thylakoid membrane structure, the thylakoid membrane protein complexes, mainly composed of PPCs, were analyzed through BN-PAGE in the leaves of ‘HJY’ and ‘FD’ with or without shading. As shown in Figure 5, the PPCs in the leaves of ‘FD’ barely changed during shading treatment, while they varied significantly in the leaves of ‘HJY’. Under natural light conditions, only a weak signal of ATPase/Cytb6f could be observed in the leaves of ‘HJY’. PSII monomeric and LHCII trimer could be detected in the leaves of the ‘HJY’ shade for 12 h. With the extension of shading time, the PSII dimer with PSI monomer, PSII monomer, PSII monomer without CP43, and LHCII assembly appeared. After being shaded for 144 h, except the weak signals of the PSII-LHCII (C_2_S), PSII-LHCII (C_2_S_2_), and PSII-LHCII (C_2_S_2_M) supercomplexes, the signals of the other complexes in ‘HJY’ increased to levels similar to the ‘FD’. 

**Figure 6:**
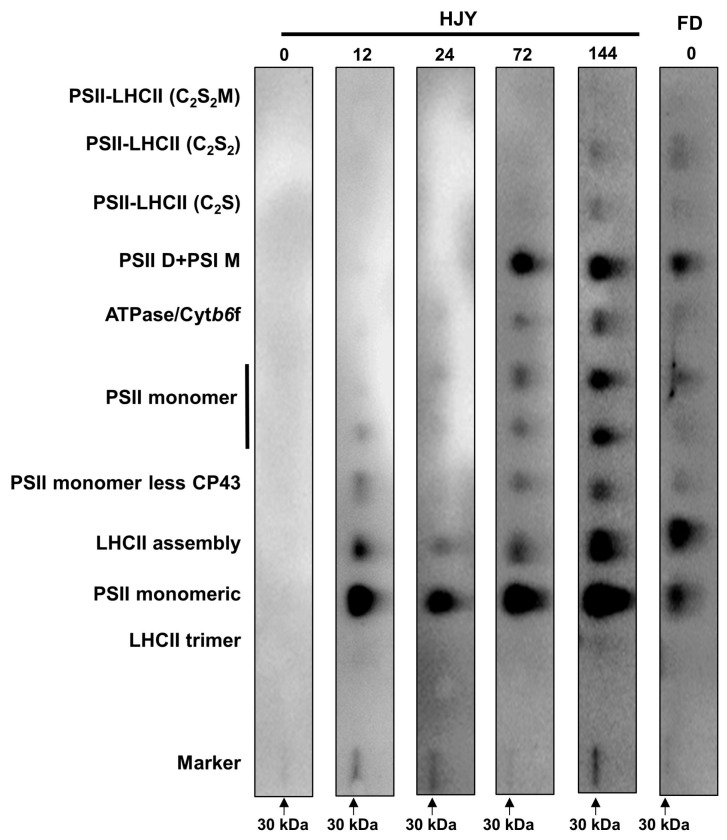
D2 signals were detected from various pigment-protein complexes in the leaves of ‘HJY’ during shading treatment. HJY0–HJY144 indicated the leaves of ‘HJY’ shaded for 0–144 h. FD0 indicated the leaves of ‘FD’ under natural light conditions.

The bands containing different PPCs were cut out and separated by the second dimension SDS–PAGE, then probed with a specific D2 antibody. As shown in Figure 6, the signal of the D2 subunit could not be detected from the PPCs in the leaves of ‘HJY’ under natural light conditions but could be carried out from the complexes of PSII monomeric, LHCII assembly, PSII monomer less CP43, and PSII monomer in the leaves shaded for 12–24 h. In addition to the complexes mentioned above, the D2 was also included in the complexes with bigger molecular weight such as PSII dimer with PSI monomer in the leaves shaded for 72 h. The signal pattern of D2 in leaves of ‘HJY’ shaded for 144 h was quite similar to that in ‘FD’ (Supplementary Figure S1). It was clear that the low level of the photosynthetic pigments and capacity might be attributed to the assembly obstacle of the PPCs in the leaves of ‘HJY’ under natural light conditions.

### 2.4. Variations of the PSII and LHCII Subunits during Shading Treatment 

**Figure 7:**
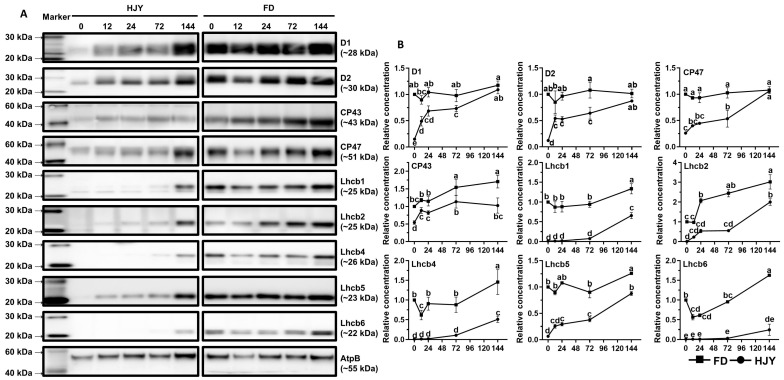
The changes in levels of the PSII core complex and LHCII subunits during short-term shading treatment. (**A**) showed immunoblot results of the target proteins; HJY0–HJY144 and FD0–FD144 indicated the leaves of ‘HJY’ and ‘FD’ shaded for 0–144 h. (**B**) showed the relative level of the target proteins, and different letters indicated the significance at *p* < 0.05 (one-way ANOVA, Duncan).

The subunits of the PSII core complex and LHCII were detected through immunoblot analysis to investigate their contribution to the albino phenotype of ‘HJY’. As shown in Figure 7, immunological signals of D1, D2, CP43, and CP47 could be detected in the leaves of ‘HJY’ under natural light conditions, but their relative concentrations were significantly lower than those in the leaves of ‘FD’ (Supplementary Figure S2). Along with the extension of shading time, the levels of the four subunits in ‘HJY’ increased significantly, whilst those in ‘FD’ changed a little. After being shaded for 144 h, the levels of D1, D2, CP43, and CP47 in ‘HJY’ increased by 6.85, 7.38, 0.88, and 3.14 folds, respectively. The levels of LHCII subunits also enhanced gradually in ‘HJY’ during the shading treatment. Except for the Lhcb5, all the LHCII subunits were too faintly weak to be detected especially in leaves of ‘HJY’ without shading or with short time shading. Meanwhile, all the LHCII subunits could be detected in all samples and changed in a roughly increased manner in FD during shading treatment. Since the chlorophylls bound to LHCII subunits accounted for nearly 50% of the total, it was clear that the albino phenotype of ‘HJY’ under natural light conditions might be directly attributed to the lack of LHCII subunits, rather than the core complex of the PSII, since deficiency of the LHCII subunits would lead to assembly obstacle of the PPCs.

### 2.5. Variation of the Gene Expression after Shading Treatment

**Figure 8:**
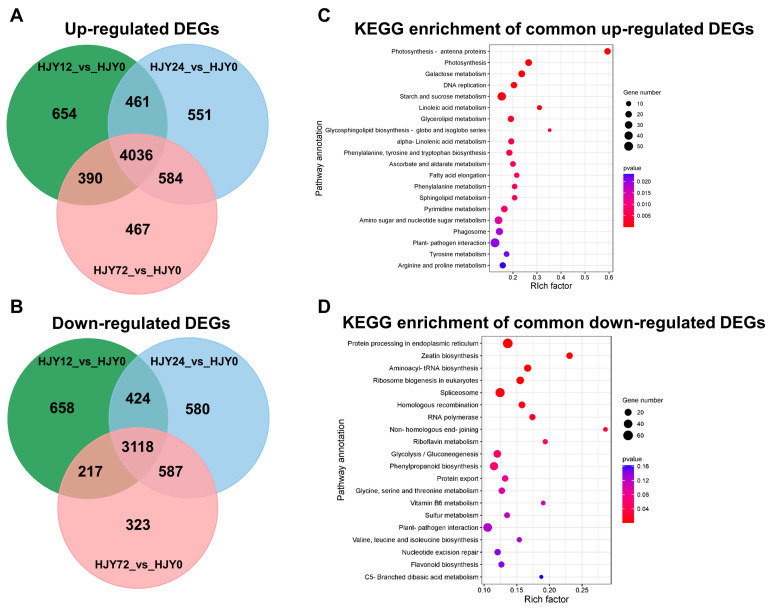
Venn diagram of the shared and unique DEGs (**A**,**B**) and KEGG pathway enrichment of the shared DEGs (**C**,**D**).

Changes in the gene expression in the second leaf of ‘HJY’ shoots shaded for different times (0 h, 12 h, 24 h, and 72 h) were analyzed through transcriptome sequencing. Each sample obtained more than 6 G data, and the Q30 base percentage was higher than 92.99% (Supplementary Table S3), indicating that the sequencing quality could meet the requirements of subsequent analysis. Based on the mapping results of clean data to the genome, the reads that could be mapped to the genes and their exon regions accounted for 84.65−87.73% and 92.39−94.72 % in each sample, respectively (Supplementary Table S4). The FPKM of each gene was calculated and used to screen the DEGs between two samples according to criterion |log_2_(fold change)| > 1 and q-value < 0.005. After comparing with the leaves under natural light conditions, 7154 DEGs were screened out from the leaves of ‘HJY’ shaded for 12–72 h using Venn diagrams analysis, in which 4036 DEGs were up-regulated and 3118 DEGs were down-regulated, respectively (Figure 8A,B).

The up-regulated DEGs were predominantly enriched in pathways associated with photosynthesis including photosynthesis antenna proteins and photosynthesis proteins, pathways involved in carbohydrate metabolism, such as galactose metabolism, starch, and sucrose metabolism, pathways related to lipid synthesis, encompassing linoleic acid metabolism, glycerolipid metabolism, glycosphingolipid biosynthesis, as well as a pathway of DNA replication (Figure 8C). This implied that shading facilitated the functional recovery of chloroplast in the leaves of ‘HJY’ by enhancing the expression of the genes associated with the synthesis or metabolism of the proteins and lipids related to the construction of photosynthetic complexes on the thylakoid membranes. Meanwhile, the down-regulated DEGs mainly belonged to the pathways related to protein synthesis and processing, gene expression, and regulation (Figure 8D), which implied that extremely activated synthesis and processing of some proteins and genes might occur in the leaves of ‘HJY’ under natural light condition and were calmed down by shading treatment. The abnormal processing might reflect the translocation and/or assembly obstacles of newly synthesized proteins and dysfunction of the target subcellular organelle(s). Among them, a large number of genes encoding the photosynthesis antenna proteins (Supplementary Figure S3) and photosynthesis (Supplementary Figure S4) were continuously up-regulated along with an extension of shading time, indicating that the expression of nuclear-encoded chloroplast proteins in the leaves of ‘HJY’ might be modulated at transcript level or post-translation level by the plastid-nucleus retrograde signaling pathway which was triggered by the shading treatment. Thus, around 50 key genes related to the pathways mentioned above in the leaves of ‘HJY’ and ‘FD’ (Supplementary Table S5) were screened out and further analyzed by qRT-PCR.

**Figure 9:**
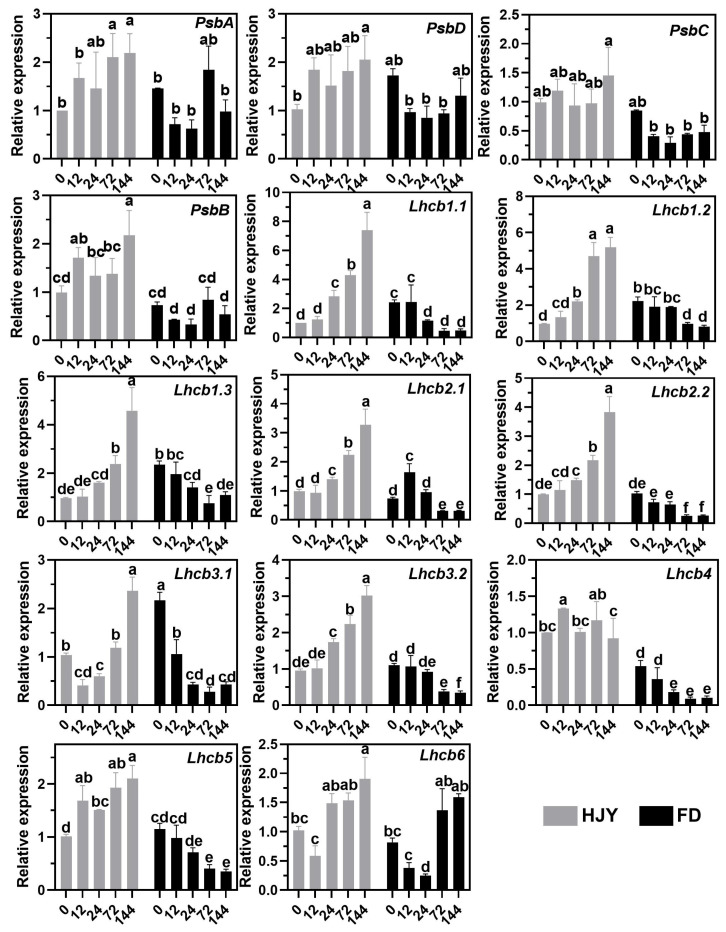
Changes in the expression level of the genes encoding the PSII and LHCII subunits in the leaves of ‘HJY’ and ‘FD’ during shading treatment (0–144 h). The horizontal axis represented the duration of shading (h) and the different letters above the columns indicated significant differences at *p* < 0.05 (one-way ANOVA, Duncan).

As shown in Figure 9, compared with the leaves of ‘HJY’ grown under natural light conditions, the relative expression levels of *PsbA*, *PsbD*, *PsbB,* and *PsbC*, which encoded the subunits of the PSII reaction center complex in the photosynthesis pathway, as well as *Lhcb1.x*, *Lhcb2.x*, *Lhcb3.x*, *Lhcb4*, *Lhcb5*, and *Lhcb6*, which encoded LHCII subunits in the photosynthesis antenna protein pathway, were all upregulated in the leaves of ‘HJY’ as shading time extended. Meanwhile, many of these genes, except the *PsbA*, *Lhcb2.1,* and *Lhcb6*, exhibited down-regulatory expression in the leaves of ‘FD’ along with the extension of the shading time, which was fully consistent with the general rule of plants’ response to the light. Interestingly, under natural light condition, only the expression of the *Lhcb1.x* in the leaves of ‘FD’ were significantly higher than that of ‘HJY’. Combined with the changes in expression of the genes related to PSII and LHCII subunits in the leaves of ‘FD’ with or without shading treatment, it was clear that suppressed expression of the LHCII subunits-encoding genes, especially the *Lhcb1.x* was closely related to the albino phenotype of the ‘HJY’.

**Figure 10:**
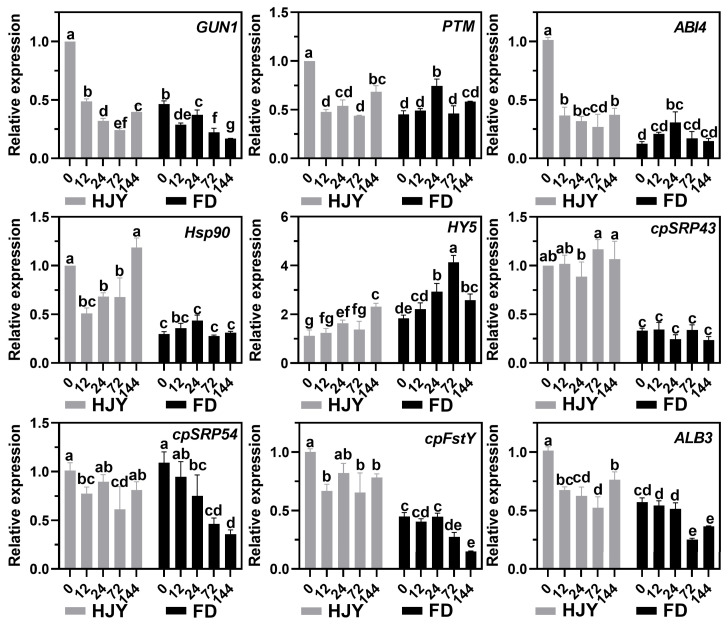
Changes in the expression level of key genes involved in plastid retrograde signaling pathway and post-translational translocation pathway in the leaves of ‘HJY’ and ‘FD’ during short-term shading. The horizontal axis represented the duration of shading (h); and different letters above the columns indicated significant differences at *p* < 0.05 (one-way ANOVA, Duncan).

Expression of the genes related to the plastid retrograde signaling pathway and LHCII subunits translocation pathway was shown in Figure 10. In the leaves of ‘HJY’ grown under natural light conditions, the expression levels of *GUN1* (*GENOMES UNCOUPLED 1*), *PTM* (*PHD type transcription factor with transmembrane domains*), *ABI4* (*ABSCISIC ACID INSENSITIVE 4*), and *HSP90* (*Heat shock protein 90*) were significantly higher than those in ‘FD’, while the expression of *HY5* (*Protein LONG HYPOCOTYL 5*) was lower than that in FD. During shading treatment, the expression levels of *GUN1*, *PTM*, and *ABI4* in ‘HJY’ leaves decreased significantly in comparison with under natural light, while a continuously and significantly up-regulated tendency was observed in the expression of the *HY5* and first decreasing and then increasing tendency was witnessed in the expression of the *Hsp90*. Meanwhile, along with the extension of shading, the expression of the GUN1 continuously down-regulated, while the expression of the PTM, ABI4, HSP90, and HY5 increased and then was followed by a decrease in leaves of ‘FD’. It was clear that the plastid retrograde signaling pathway GUN-PTM-ABI4 was abnormally regulated in the leaves of ‘HJY’ compared with that in ‘FD’, suggesting that this signaling pathway might be involved in the regulation of the albino phenotype of ‘HJY’ because these genes’ products could usually modulate the LHC subunit-related gene expression directly or indirectly.

Although the expression levels of the LHC subunits translocation and thylakoid insertion-related genes cpSRP43 (43 kD chloroplast signal recognition particle), cpFstY (Cell division protein FtsY homolog), and ALB3 (Inner membrane protein ALBINO3) in the leaves of ‘HJY’ under natural light condition were significantly higher than those in ‘FD’, changes in the expression levels of these genes along with the extension of shading time were quite similar in the two cultivars. This indicated that the expression of the genes related to the translocation into chloroplast and insertion into the thylakoid membrane of the LHC subunits was remarkably induced by low expression of LHCs subunit-encoding genes through a positive feedback manner under natural light conditions. In other words, the high expression of the cpSRP43, ALB3, and cpFtsY seemed to be a response against the shortage of LHC subunits, but not to be the direct cause of the albino phenotype in ‘HJY’.

## 3. Discussion

### 3.1. Normalization of Leaf Color and Function Needed Thylakoid Structure Recovery

Under natural light conditions, the newly spouted shoots of the light-sensitive albino tea cultivar ‘HJY’ exhibited a pale-yellow phenotype, their Fv/Fm values and the contents of photosynthetic pigments were significantly lower than those of the normal green cultivar ‘FD’, and exhibited abnormal cellular ultrastructure including a reduced number of chloroplasts as well as a lack of grana thylakoids but an increased voids in the chloroplast (Figure 1, Figure 2, Figure 3 and Figure 4). These findings were consistent with previous studies on photosynthesis efficiency [13], pigments [5,11], and ultrastructure [5] of the ‘HJY’. When the ‘HJY’ was grown under low light conditions through shading, the leaf color, photosynthesis efficiency, pigments content, and chloroplast architecture gradually turned to normalness along with the extension of the shading time (Figure 1, Figure 2, Figure 3 and Figure 4). It could be speculated that the coding region of the genes related to photosynthesis, pigments biosynthesis, and chloroplast ultrastructure might be normal in ‘HJY’ since low light treatment even for a short period could remarkably reverse the abnormal phenotype of this cultivar.

Chlorophylls could absorb, transfer and convert the light energy through binding to PSII center subunits and light-harvesting complexes during the primary reaction and photoreaction stages, however, a large number of free radicals might also be generated by the photo-excited chlorophylls during this process, which would destroy the chlorophylls themselves and influence the photosynthetic apparatus stability [16]. Carotenoids, another important type of pigment component in the leaves, could harvest and transfer light energy to the reaction center through binding with LHCII subunits [17] but also could quench the free radicals and prevent the photooxidation of the chlorophylls and proteins through the xanthophyll cycle [17,18]. In this study, the levels of lutein and violaxanthin changed in a contrary tendency, decreased lutein, and increased violaxanthin, during the shading treatment. This implied that the leaves of ‘HJY’, under natural light conditions, suffered from extreme photooxidation damage indicated by the low level of violaxanthin and high level of lutein [17]. Therefore, low accumulation of chlorophylls might be a self-protection mechanism in the albino leaves of ‘HJY’ because the free chlorophylls were usually dangerous to photosynthetic apparatus for their radical-inducing potentials. The thylakoid membrane, as a key site in the light reaction stage of photosynthesis, could accomplish the transferal of electrons from H_2_O to NADP^+^ and the production of ATP while releasing O_2_ through a variety of embedded transmembrane PPCs [19,20]. Among them, PSI and PSII were critical for biological light energy conversion and photosynthesis efficiency [21]. Chlorophyll fluorescence kinetic curves and the relevant parameters offered a comprehensive reflection of plant growth, stress occurrence, and the degree of PSII photoinhibition [22]. According to our observation, the recovery of the chloroplast structure, in particular, some parameters of the thylakoids and plastoglobules was earlier than that of chlorophylls and photosynthetic parameters in shaded leaves of ‘HJY’, indicating that restoration of the thylakoid structure might be the prerequisite for normalization of the leaf color and function since only an integrated thylakoid structure could help to the efficient synthesis and full accumulation of chlorophylls and the effective improvement of photosynthetic parameters.

### 3.2. Proper Assembly of PPCs Needed for the LHCII Subunits

Much research on the mechanisms of the albino phenotype formation had primarily focused on pigment metabolism pathways [7] since the mutants exhibited obvious chlorosis symptoms. However, numerous DEGs belonged to the photosynthesis-related pathways although the content of photosynthetic pigments also increased in the shaded leaves of ‘HJY’ [8]. Furthermore, high light exposure had been found to accelerate the depolymerization of PSII complex subunits such as CP43, CP47, PsbP, and PsbR, resulting in the degradation of the reaction center proteins D1 and D2 [13]. These findings suggested that the albino phenotype of tea plants might be closely associated with the abnormal assembly of the photosynthesis-related proteins in addition to the lack of pigments [9,23]. In the leaves of ‘HJY’ under natural light condition, PSII subunits D1, D2, CP43, and CP47 could be detected in very low levels from the total chloroplast proteins, however, the D2 signals could not be conducted from the PPCs on the thylakoid membrane. Along with the extension of shading time, the levels of D1, D2, CP43, and CP47 in ‘HJY’ leaves were significantly increased, and D2 signals gradually appeared in the PPCs, such as PSII monomeric, LHCII assembly, PSII monomer without CP43, and PSII monomer (Figure 5, Figure 6 and Figure 7). These findings suggested that the reaction center subunits of the PSII mainly existed as free monomers in the leaves of ‘HJY’ under natural light conditions, but could be assembled into PPCs in the shaded leaves. Therefore, the albino phenotype of ‘HJY’ might be attributed to the assembly obstacle of the PPCs on the thylakoid membrane instead of a lack of pigments.

According to our observation, Lhcb1, Lhcb2, Lhcb4, and Lhcb6 subunits as well as the LHCII trimers and LHCII-containing protein complexes could hardly be detected in the leaves of ‘HJY’ under natural light conditions, but could gradually be conducted in the shade leaves. The low levels of the LHCII subunits, in particular, the extremely low Lhcb1 resulted in a deficiency of LHCII trimer and other LHCII-containing protein complexes in the albino leaves of ‘HJY’. The report showed *Arabidopsis thaliana Lhcb2* deletion mutant (*lhcb2*) only contained about 60% of chlorophylls of the wild type [24]. Overexpression of a tea plant LHCII subunit encoding gene CSA035910 could rescue light-green phenotype of the homologue-deleted *A. thaliana* and enhance the leaf chlorophyll accumulation, suggesting expression of the LHCII subunit genes had a close relationship with the chlorophyll biosynthesis and accumulation [23]. The LHCII trimer had been proven to consist of Lhcb1, Lhcb2, and Lhcb3 subunits in higher plants. When the Lhcb2 was replaced with Lhcb1, *A. thaliana* contained similar thylakoid PPCs to the wild-type plants, conversely, when the Lhcb1 was replaced with Lhcb2, the plants exhibited significant antenna remodeling and thylakoid membrane structure variation due to the reduced LHCII trimers [25]. Therefore, the severe deficiency of Lhcb1 might be one of the most important factors responsible for the assembly of thylakoid PPCs in the leaves of ‘HJY’. Shortage of Lhcb1 would lead to assembly failure of the LHCII trimer which could not only bind a large amount of pigments but also stabilize the LHCII-containing protein complexes [26].

### 3.3. Low-Level Expression of the Lhcbs Was Modulated by GUN1-PTM-ABI4

Gene expression analysis showed that numerous DEGs were enriched in the photosynthesis and photosynthesis-antenna proteins during shading treatment (Figure 8). Expressions of the key photosynthetic protein genes were up-regulated in the leaves of ‘HJY’ after being shaded. On the contrary, expression of the genes encoding the PSII reaction center core complex and LHCII subunits were gradually and significantly suppressed along with the extension of the shading time in the normal green cultivar ‘FD’ (Figure 9), which was consistent with the previous research on model plants [27]. Thus, the expression pattern of the photosynthesis-related genes was significantly different in the leaves of ‘HJY’ and ‘FD’ during shading treatment. It was clear that the low level of the LHCII subunits in the albino leaves of ‘HJY’ grown under natural light was due to the suppressed expression of the *Lhcb* genes, especially the *Lhcb1.x*.

**Figure 11:**
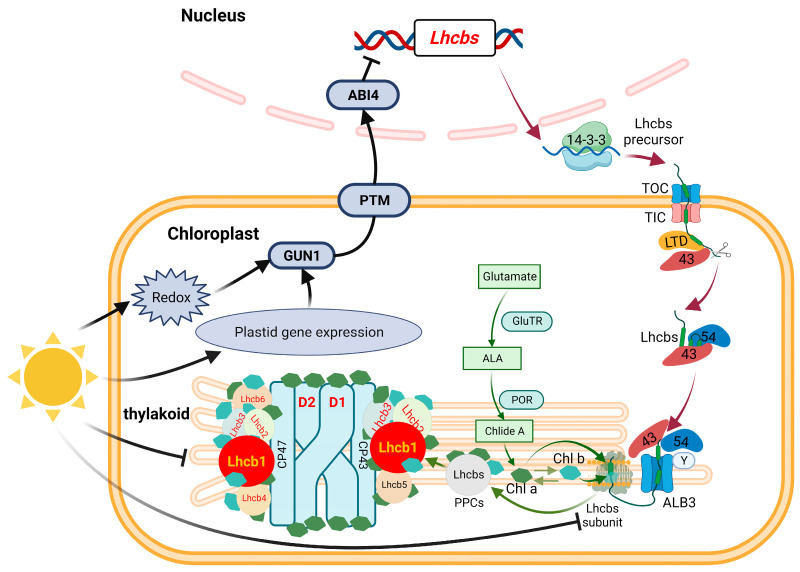
The Albino phenotype of ‘HJY’ might be modulated by a plastid retrograde signaling pathway. The albino phenotype of ‘HJY’ grown under natural light might be mainly attributed to the abnormal assembly of the thylakoid structure, which was induced by the lack of LHCII subunits, especially the Lhcb1. The low level of LHCII subunits was due to the suppressed expression of the relevant genes which was modulated by the GUN1-PTM-ABI4 pathway. The black arrow (→) indicated the acceleration, the black blunt line (┤) indicated the inhibition and other arrows indicated the metabolism processing or delivery pathway. 14-3-3, 14-3-3 domain-containing protein; GUN1, GENOMES UNCOUPLED 1; PTM, PHD type transcription factor with transmembrane domains; ABI4, ABSCISIC ACID INSENSITIVE 4; TOC, Translocon at the outer envelope membrane of chloroplasts; TIC, Translocon at the inner envelope membrane of chloroplasts; LTD, Light-harvesting complex translocation deficient; 43, Chloroplast signal recognition particle 43; 54, Chloroplast signal recognition particle 54; Y, Chloroplast signal recognition particle Y; ALB3, Albino 3; GluTR, Glutamyl-tRNA reductase; ALA, 5-aminolevulinic acid; POR, Protochlorophyllide oxidoreductase; Chl a, Chlorophyll a; Chl b, Chlorophyll b.

In photosynthetic proteins, apart from the PS reaction center proteins encoded by chloroplast genes, most of the PS and LHC complex subunits were nuclear-encoded, synthesized in the cytoplasm, and then transported into chloroplasts through post-translational translocation mechanisms [17,28,29]. LHC protein subunits were imported into chloroplasts by forming transportation complexes with cpSRP heterodimers and inserted into thylakoid membranes with the assistance of the cpFtsY and Alb3 [30]. Mutation in translocation-related genes would lead to a significant reduction in levels of LHC subunits and pigments, resulting in pale green or bleached phenotypes [31]. In higher plants, chloroplast development was largely regulated by the nuclear genes, but could also influence the nuclear gene expression through retrograde signaling pathways. These pathways transmitted the physiological and metabolic status information of the chloroplasts to the nucleus via a series of signal molecules and transduction styles, particularly when chloroplast development was arrested or blocked [32,33]. According to the comparison of the expression of the genes related to retrograde signaling transduction pathways and post-translational transport pathways [33,34], the GUN1-PTM-ABI4 retrograde signaling pathway might be responsible for the regulation of the *Lhcbs* expression. These signal-related genes were highly expressed in the leaves of ‘HJY’ under natural light but significantly down-regulated in shaded leaves (Figure 10). GUN1, a pentatricopeptide repeat domain-containing protein encoded by *GENOMES UNCOUPLED 1*, was located in the chloroplast nucleoid region [35]. PTM, a membrane-bound transcription factor, was situated on the chloroplast outer membrane. When PTM received the chloroplast signal, it would be released from the membrane, and entered into the nucleus, then activated the ABI4 transcription through binding to the ABI4 promoter region [36]. ABI4, an AP2-type ethylene-responsive transcriptional regulator, could respond to chloroplast signals and suppress the *Lhcbs* transcription through competitive binding to the S-BOX sequence element (5’-CATTCCA-3’) in the promoter region of the *Lhcbs* [37]. Therefore, the albino phenotype of ‘HJY’ under natural light might be mainly related to the abnormal assembly of the thylakoid structure which was induced by the lack of LHCII subunits (especially the Lhcb1), and the low expression of *Lhcbs* might be attributed to the high expression of GUN1-PTM-ABI4 signaling pathway-related genes (Figure 11). As GUN1 could respond to multiple pathways mediated by tetrapyrrole metabolic intermediates, chloroplast gene expression, and reactive oxygen species [35,38,39,40], further research should be conducted to identify the exact GUN1-responding signals or factors released from the abnormal chloroplast in the leaves of ‘HJY’ under natural light condition. In addition, the expression pattern of the post-translational translocation-related genes was generally similar in the leaves of ‘HJY’ and ‘FD’ during the shading although the expression level of these genes in ‘HJY’ leaves were higher than those in ‘FD’ under natural light. This implied that the post-translational translocation-related genes might not be involved in the albino phenotype formation of the ‘HJY’.

## 4. Materials and Methods

### 4.1. Plant Materials and Chemicals

The light-sensitive albino tea cultivar ‘Huangjinya’ (‘HJY’) and the normal green tea cultivar ‘Fudingdabaicha’ (‘FD’) were planted in the experimental tea garden of Zhejiang University (latitude: 30.28° N, longitude: 120.15° E, Hangzhou, China). These trees were 5 years old and well maintained through common agronomic practices under the same soil, altitude, and climate conditions.

Chlorophyll a, chlorophyll b, lutein, violaxanthin, neoxanthin, β-carotene, and cross-linked polyvinylpolypyrrolidone (PVPP)were purchased from Sigma-Aldrich Corporation (St. Louis, MO, USA). Analytical grade acetone, anhydrous ethanol, quartz sand, glutaraldehyde, phosphoric acid, and osmium acid were purchased from Sinopharm Chemical Reagent Co., Ltd. (Shanghai, China). Chromatographic grade acetonitrile, methanol, acetic acid, and chloroform were obtained from SK Global Chemical Co. (Seoul, Republic of Korea). RNAprep pure Plant Kit was purchased from TIANGEN Biotech Co., Ltd. (Shanghai, China). HiScript™ II Q RT SuperMix Kit and ChamQ SYBR qPCR Master Mix Kit (High ROX Premixed) were purchased from Vazyme Biotech Co., Ltd. (Nanjing, China). Minute^TM^ Chloroplast Isolation Kit and denatured protein solution were obtained from Invent Biotechnologies, Co., Ltd. (Plymouth, MA, USA). BCA Protein Quantification Kit was purchased from Yeasen Biotech Co., Ltd. (Shanghai, China). SDS sample buffer, 4 × LDS sample buffer, and 4~12% precast resolving sodium dodecyl sulfate-polyacrylamide gel electrophoresis (SDS-PAGE) were ordered from GenScript Biotech Co., Ltd. (Nanjing, China). N-dodecyl-β-D-maltoside (β-DM), Serva Blue G-250, and Blue Native Polyacrylamide Gel Electrophoresis (BN-PAGE) Kit were obtained from Realtimes Co., Ltd. (Beijing, China). Unless otherwise stated, all other reagents were obtained from Sangon Biotech Co., Ltd. (Shanghai, China).

### 4.2. Shading Treatment

The shading treatment was performed on the bushes of the two cultivars for 0–6 days from 21 to 27 April 2022 (maximum temperature: 26.2 ± 3.0 °C; minimum temperature: 17.6 ± 2.82 °C). In detail, 10 bushes for each treatment were covered at 2:00 p.m. on 21, 24, and 26 April and at 2:00 a.m. on 27 April with the triple-layer high-density black polyethylene net (Shuyang Flower Gardening Co., Ltd., Jiangsu, China) all around, respectively. The distance between the plucking table of the bushes and the shading net was about 30 cm. The light intensity on the plucking table was measured daily using a model 1332A digital lux meter (TES Electrical Electronic Corp., Taibei City, China), and the average intensity at 2:00 p.m. was 166.46 ± 13.12 μmol photons m^−2^ s^−1^ (Supplementary Table S1), with a light transmittance of 10.66 ± 0.89% compared with natural light condition (average intensity at 2:00 p.m.: 1563.58 ± 54.02 μmol photons m^−2^ s^−1^). Sampling was carried out at 2:00 p.m. on 27 April. Around 50 g tender leaves (the 2nd leaf down the apical bud) were randomly harvested after the different time shading treatments and designated as HJY144, HJY72, HJY24, and HJY12 for the cultivar ‘HJY’ and as FD144, FD72, FD24, FD12 for the cultivar ‘FD’, respectively. As a control, tender leaves similar to the treatment were also picked up at 2:00 p.m. on 27 April from the bushes without any shading and designated as HJY0 and FD0. All the treatment was repeated three times. The collected samples were immediately frozen in liquid nitrogen and stored at −80 °C for further analysis unless otherwise specified.

### 4.3. Measurement of Chlorophyll a Fluorescence Transient

The chlorophyll fluorescence transient was determined using a pulse amplitude modulation fluorimeter Handy PEA (Hansatech Instruments, Norfolk, UK) according to the instrument operation manual. The 30 min dark-adapted leaves were excited by an array of three light diodes peaking at 650 nm and a photon flux density of 3000 μmol photons m^−2^ s^−1^, and fluorescence was detected by PIN-photodiode. The chlorophyll fluorescence kinetics was measured from 10 μs to 1 s, and “O” was used to refer to the origin fluorescence (Fo) at 0 ms, “J” the junction fluorescence at 20 ms, “I” the inflection fluorescence at 30 ms, and “P” the maximal fluorescence (Fm). The curves of the “OJIP” were normalized as relative variable fluorescence. Fv/Fm, the key parameter of “OJIP” was determined as the previous method [15] with 12 leaves (the third leaf down the apical bud on the shoot) in each treatment, and an average value was used for calculation.

### 4.4. Analysis of Photosynthetic Pigments

Leaf discs without main veins (200 mg) were grounded with liquid nitrogen and mixed with 10 mL pre-cooled acetone and 50 mg PVPP, and then ultrasonically extracted for 30 min in a dark ice-water bath (JP-060S, Skymen Cleaning Equipment Shenzhen Co., Ltd., Shanghai, China). The mixture (1.2 mL) was transferred into a centrifugal tube (1.5 mL) and centrifuged at 12,000 rpm and 4 °C for 15 min. The supernatant was transferred into a brown high-performance liquid chromatography (HPLC) vial. Analysis of photosynthetic pigments, including chlorophyll a, chlorophyll b, neoxanthin, violaxanthin, lutein, and β-carotene was performed on an LC-20AT HPLC System coupled with a UV-vis detector (Shimadzu, Kyoto, Japan). A TC-C_18_ column (5 μm, 4.6 × 150 mm; Agilent Technologies Inc., Santa Clara, CA, USA) was used to separate the pigments. HPLC conditions were as follows: column temperature, 35 °C; injection volume, 10 μL; mobile phase A, 3% acetonitrile + 0.5% acetic acid + 96.5% water (*v*/*v*); mobile phase B, 75% acetonitrile + 20% methanol + 5% chloroform (*v*/*v*); linear gradient elution program, mobile phase B increasing from 90% to 100% in the early 15 min, holding at 100% for another 15 min, then back to 90% in 5 min; total flow rate, 1 mL min^−1^; detection wavelength, 440 nm. The detected pigments were qualified and quantified by comparing them with the retention time and peak area of the authentic reference compounds.

### 4.5. Transmission Electron Microscopy (TEM) Observation

The freshly harvested leaf was cut into small discs (1–2 mm^2^) and fixed with 2.5% glutaraldehyde in 0.1 M phosphate buffer (pH 7.0) at 4 °C overnight, then washed with 0.1 M phosphate buffer (pH 7.0) three times. The leaf discs were post-fixed with 1% osmium tetroxide in 0.1 M phosphate buffer (pH 7.0) for 1 h at room temperature and washed with 0.1 M phosphate buffer (pH 7.0) three times, and then dehydrated by a gradient ethanol solution (30, 50, 70, 80, 90, 95, and 100%, *v*/*v*) each for 15 min and by 100% acetone for 20 min at room temperature. The dehydrated discs were embedded in a mixture of acetone and Spurr resin (1:1, *v*/*v*) for 1 h, then transferred in a 1:3 (*v*/*v*) mixture of acetone and Spurr resin, and finally in pure Spurr resin overnight at room temperature. The embedded sample was sectioned by using LEICA EM UC7 ultratome (Leica, Schott, Germany), and the sections were stained with uranyl acetate and alkaline lead citrate for 5–10 min, respectively, and observed on a Hitachi Model H-7650 TEM (Hitach Ltd., Tokyo, Japan).

### 4.6. Analysis of the PSII and LHCII Subunits

Chloroplast proteins were isolated from the leaves of the ‘HJY’ and ‘FD’ shaded for different times (0, 12, 24, 72, and 144 h) with the Minute^TM^ Chloroplast Isolation Kit according to the manufacturer’s instructions, and were dissolved in the Minute™ denatured protein solution. The concentration of the proteins was measured by using BCA Protein Quantification Kit and was automatically calculated using a Nanodrop spectrophotometer (Thermo Scientific, Waltham, MA, USA). The concentration of the chloroplast proteins was adjusted to 6.67 mg mL^−1^ using the denatured protein solution. Afterward, the proteins were denatured in 4 × LDS sample buffer at 70 °C for 10 min, the denatured proteins were separated on a 4~12% precast resolving SDS-PAGE and then transferred onto the nitrocellulose membranes via semi-dry transfer with a transblot apparatus (Bio-Rad, Hercules, CA, USA). The proteins were probed with the primary antibodies raised in rabbits purchased from Agrisera (Vännäs, Sweden) for immunoblot detection. The dilution ratios of the primary antibodies used were as follows: for PSII—D1 (1:10,000), D2 (1:5000), CP43 (1:3000), CP47 (1:2000); for LHCII—Lhcb1 (1:2000), Lhcb2 (1:5000), Lhcb4 (1:7000), Lhcb5 (1:1000), Lhcb6 (1:5000); and AtpB (1:5000). In this case, the antibody against AtpB was used as a control for standardizing the loading amount of different samples.

### 4.7. Analysis of the Pigment-Protein Complexes (PPCs)

PPCs were isolated from the leaves of the ‘HJY’ and ‘FD’ shaded for different times (0, 12, 24, 72, and 144 h) using the Minute^TM^ Chloroplast Isolation Kit with modification according to Aro et al. [41] and Nama et al. [42]. Leaf discs without main veins (200 mg) were ground with liquid nitrogen and transferred into the filter of the kit, and then 100 μL cold buffer A and 50 mg PVPP were added. The mixtures were ground with a plastic rod about 100 times. Another 300 μL buffer A was added and mixed thoroughly by stirring with the pipette tip. The mixtures were centrifuged at 2000× *g* at 4 °C for 5 min. The filter was discarded and the supernatant in the collection tube was removed. The obtained pellets in the collection tube were re-suspended in 200 μL of the cold shock buffer (50 mM Hepes-KOH, 5 mM MgCl_2_, 10 mM NaF, pH 7.5) and incubated on the ice for 10 min. The mixtures were then centrifuged at 20,000× *g* at 4 °C for 20 min. The pellets containing the purified thylakoid membrane were collected after the removal of the supernatant. The pellets were re-suspended in 15–30 μL of cold storage buffer (50 mM Hepes-KOH, 100 mM sorbitol, 10 mM MgCl_2_, 10 mM NaF, 10 mM NaCl, pH 7.5). The concentration of the total chlorophylls was measured at 645 nm and 663 nm on a NanoDrop spectrophotometer, and calculated using the equation suggested by reference [43], and then adjusted to 0.3 mg mL^−1^ with the cold sample buffer (25 mM BisTris-HCl, 20% (*w*/*v*) glycerin, pH 7.0). An equal volume of the sample buffer containing 2% (*w*/*v*) β-dodecyl maltoside (β-DM) was added to the purified thylakoid membrane in the buffer. The mixture was gently flicked with fingers until the suspension became clear, and then incubated on ice in the dark for 10 min. Subsequently, the mixture was centrifuged at 18,000× *g* at 4 °C for 20 min, and the supernatant containing the dissolved PPCs was collected. Finally, a mixture containing 13 μL of PPCs solution, 5 μL of BN-PAGE sample buffer (50 mM BisTris-HCl, 50 mM NaCl, 10% (*w*/*v*) glycerol, pH 7.2), and 2 μL of Coomassie Brilliant Blue G-250 buffer (5% (*w*/*v*) Serva Blue G-250) was prepared for subsequent BN-PAGE separation. The mixtures were loaded on a non-denaturing BN-PAGE for the first dimension separation according to the Kit manufacturer’s instructions. Bands of PPCs were identified according to the methods described by Aro et al. [41] and Dhokne et al. [44]. Before the second dimension SDS–PAGE, gel pieces containing PPCs with different molecular weights were cut out from the same lane of the BN-PAGE gel, respectively, and then denatured in the SDS sample buffer at room temperature for 30 min. The denatured proteins were loaded on the 4~12% precast resolving SDS-PAGE gel for the second dimension separation as mentioned above. The D2 protein was detected through immunoblot analysis using the primary antibody against D2 (Agrisera). 

### 4.8. Transcriptomic Analysis and qPCR Confirmation

Total RNAs were isolated from the leaves of ‘HJY’ and ‘FD’ shaded for different times (0, 12, 24, and 72 h) using the RNAprep pure Plant Kit according to the manufacturer’s instructions. Subsequently, the concentration, quality, and integrity of RNA were determined using a NanoDrop spectrophotometer (Thermo Fisher Scientific, Waltham, MA, USA). The cDNA libraries were constructed by using the NEBNext Ultra RNA Library Prep Kit for Illumina (NEB, Ipswich, MA, USA) and then sequenced on Illumina Hiseq^TM^ 2500 platform. The obtained clean reads were mapped to the reference genome sequence of *C. sinensis* cv. Shuchazao (http://tpdb.shengxin.ren/, accessed on 1 November 2021) using HISAT2 (v2.0.5). The value of fragments per kilobase per million mapped reads (FPKM) was used as the expression abundance of each gene [45], and the analysis of DEGs between two samples was conducted using the DEGseq software (v1.20.0) with the criterion of |log_2_(fold change)| > 1 and significant *q*-value < 0.005. The genes were annotated based on the NR, Pfam, Swiss-Prot, KEGG, and GO databases by using the BLASTALL package with the significant threshold of *E*-value ≤ 10^−5^. ClusterProfiler software (v3.4.4) was used to carry out the enrichment analysis of the KEGG pathway of the DEGs. The transcriptomic analysis was repeated three times. Total RNAs were isolated from leaves of the ‘HJY’ and ‘FD’ shaded for different times (0, 12, 24, 72, and 144 h), and were reversely transcribed into first-strand cDNA using the HiScript™ II Q RT SuperMix kit. Subsequently, cDNAs were used as templates for the qRT-PCR. The reaction solution was prepared according to the protocol of the ChamQ SYBR qPCR Master Mix kit. Amplification was performed on the StepOnePlus™ Real-Time PCR System (ABI, Carlsbad, CA, USA), and cycling conditions were as follows: denaturing at 95 °C for 2 min, 1 cycle; denaturing at 95 °C for 3 s, annealing and extension at 60 °C for 30 s, 40 cycles. *β-Actin* was used as an internal control. All primer sequences were shown in Supplementary Table S2. Gene expression analysis was performed in triplicate for each sample. The relative level of gene expression was calculated according to the equation of 2^−ΔΔCt^.

### 4.9. Statistical Analysis

Data collation was performed using Microsoft Excel 2016 (Microsoft Corporation, Washington, WA, USA), and statistical analysis was conducted on SPSS Statistic 21.0 (IBM, Armonk, NY, USA). One-way ANOVA and Duncan’s post-hoc test were used for significant difference analysis, and *p* < 0.05 was considered to be significantly different.

## 5. Conclusions

The content of photosynthetic pigments, photosynthesis efficiency, and chloroplast ultrastructure could be normalized in the leaves of the light-sensitive albino tea plant ‘HJY’ after being shaded. The albino phenotype of the ‘HJY’ under natural light might be attributed to the disrupted assembly of thylakoid structure instead of lack of the pigment synthesis capability. The protein level of the PSII reaction center core complex, LHCII subunits, and PPCs in the leaves of ‘HJY’ increased significantly with an extension of the shading time. The abnormal assembly of the thylakoid structure might be due to the deficiency of stable PPCs which was induced by extremely low levels of the LHCII subunits, in particular, the Lhcb1. Up-regulated expression of the genes related to photosynthesis such as *Lhcbs* was observed in the leaves of ‘HJY’ during shading treatment, indicating the *Lhcbs* were suppressed in the albino leaves of ‘HJY’ under natural light. Expression of *Lhcbs* was mainly modulated by plastid retrograde signaling pathway GUN1-PTM-ABI4.

## Figures and Tables

**Table 1:** The changes in parameters of the chloroplast and its subcellular organelles in ‘HJY’ leaves during short-term shading treatment.

Item	HJY0	HJY12	HJY24	HJY72	HJY144
Chloroplast area (µm^2^)	3.80 ± 1.82 ^b^	4.52 ± 1.46 ^ab^	5.44 ± 1.85 ^a^	4.36 ± 1.61 ^ab^	4.59 ± 1.81 ^ab^
Thylakoid thickness (µm)	0.000 ± 0.000 ^c^	0.000 ± 0.000 ^c^	0.045 ± 0.001 ^b^	0.112 ± 0.032 ^ab^	0.197 ± 0.057 ^a^
Number of thylakoids in Chloroplast	2.75 ± 1.71 ^c^	14.44 ± 7.11 ^b^	29.70 ± 14.24 ^a^	30.80 ± 7.28 ^a^	29.90 ± 7.77 ^a^
Thylakoid area (µm^2^)	0.030 ± 0.036 ^ab^	0.014 ± 0.011 ^b^	0.014 ± 0.005 ^b^	0.037 ± 0.021 ^ab^	0.067 ± 0.025 ^a^
Thylakoid area density (%)	0.94 ± 0.63 ^c^	4.44 ± 2.89 ^c^	7.64 ± 3.78 ^c^	25.12 ± 12.15 ^b^	43.83 ± 9.84 ^a^
Number of plastoglobules in chloroplast	16.00 ± 13.72 ^ab^	26.60 ± 20.30 ^a^	10.70 ± 5.62 ^b^	22.50 ± 10.87 ^ab^	13.67 ± 8.56 ^b^
Plastoglobule area (µm^2^)	0.001 ± 0.001 ^c^	0.005 ± 0.003 ^ab^	0.007 ± 0.003 ^a^	0.004 ± 0.002 ^b^	0.007 ± 0.004 ^a^
Plastoglobule area density (%)	0.25 ± 0.08 ^b^	2.27 ± 0.92 ^a^	2.31 ± 1.08 ^a^	2.24 ± 1.20 ^a^	1.84 ± 1.06 ^a^

Note: Area density referred to the area percentage of the specific subcellular organelles vs. the chloroplast. HJY0–HJY144 indicated ‘HJY’ leaves shaded for 0–144 h. Different letters indicated the significance at *p* < 0.05 (one-way ANOVA, Duncan).

## Data Availability

Data are available on request from the corresponding author.

## Supplementary Materials

The supporting information can be downloaded at: https://www.mdpi.com/article/10.3390/ijms241210314/s1.
