# Supplementary material for: Shading-Dependent Greening Process of the Leaves in the Light-Sensitive Albino Tea Plant ‘Huangjinya’: Possible Involvement of the Light-Harvesting Complex II Subunit of Photosystem II in the Phenotypic Characteristic
Source: Int J Mol Sci. 2023 Jun 18;24(12):10314. doi: 10.3390/ijms241210314 (PMC10298860; doi:10.3390/ijms241210314)
Supplement: Supplementary file 1 [file ijms-24-10314-s001.zip › ijms-2433626-supplementary.pdf]

Supplementary files

Table S1. Light intensity on the plucking table of tea bush under natural light condition and after shading treatment (μmol photons m^{-2} s^{-1})

Time	Natural light	Shading treatment	Light transmittance (%)
14:00, 21th	1528.50±28.56	160.00±10.13	10.46±0.53
14:00, 24th	1603.17±34.44	161.67±15.73	10.08±0.91
14:00, 26th	1589.17±30.16	170.50±10.69	10.74±0.78
14:00, 27th	1533.50±66.35	173.67±9.55	11.35±0.78
Average	1563.58±54.02	166.46±13.12	10.66±0.89

Table S2. Sequences of primer pair for quantitative real-time PCR

Gene ID	Gene name	Primer sequence（5'-3'）
LOC114296039	*β-actin*	F: cttcctcatgctatcctccgtctt; R: atttcccgttcagcagtggtg
LOC14412515	*PsbA*	F: tcggcttttaagtgcggcta; R: tccccttcaggatcagtcgt
LOC114319249	*PsbB*	F: cagctgcccggagtctattt; R: tacgaatctcctcagcacgc
CSS0037852	*PsbC*	F: acctgcgtgcctatgacttc; R: cgccatccaagcacgaatac
CSS0011377	*PsbD*	F: tggctccaactgggttttgt; R:cgatgacaaacggaagtcgc
CSS0013089	*Lhcb1.1*	F: cgttggcagaggaaggatca; R: cgaacttgacaccattgcgg
CSS0039893	*Lhcb1.2*	F: Gaaccgtgagcttgaggtga; R: gctccagccttgaaccagat
CSS0010537	*Lhcb1.3*	F: taggagaggtgacagacccg; R: ccaggttctccaatggtccc
CSS0017867	*Lhcb2.1*	F: tgtcaagttcggtgaggcag; R: attgccagccgtccattctt
CSS0046252	*Lhcb2.2*	F: tccaaacctgatccatgccc; R: ggcctggacgaagaatccaa
CSS0015941	*Lhcb3.1*	F: gccagtatggttcaaagccg; R: ggtcatcggcaagacccaat
CSS0049576	*Lhcb3.2*	F: ccctggtgactatggttggg; R: ttgagctcctgccttgaacc
CSS0043476	*Lhcb4*	F: catgttggctacgcttggtg; R: tccggggtacaacctcttct
LOC114267048	*Lhcb5*	F: aggcggtccatttgatccat; R: gcaagcacagtgagcaagtt
CSS0010636	*Lhcb6*	F: agtcgcagtctgttgagtgg; R: gtcttcccttgtccagcctc
CSS0010117	*GUN1*	F: gcatttcggccgccaaaa; R: atcgccgccactgtatcg
CSS0018354	*PTM*	F: gcagaaaacgtgctcggc; R: tcccacccctccaccaaa
CSS0005165	*ABI4*	F: gtaaacgggtcgggctcc; R: tttcgccgccgtcttctt
CSS0018705	*Hsp90*	F: ccgtcttgctgacacccc; R: gagcaccctcttgcctcg
CSS0027798	*ALB3*	F: ccactggcagggtgtctg; R: agttggaccagcgaggga
LOC114264785	*cpSRP43*	F: ggacggtgcagataacgagt; R: actccgccacagcatattcc
CSS0014332	*cpSRP54*	F: aggcagtttgcgggactc; R: tgttgccatgtccgctga
CSS0006849	*cpFtsY*	F: cggtcatcgacgagctcc; R: cccgcaagctctccacaa
LOC114318181	*HY5*	F: ggagagtgccggagatcg; R: gaagatgcctggacccgg

Table S3. RNA sequencing data obtained from the leaves of ‘Huangjinya’ during short-term shading

Name	Sequence number	Bases number	Q30 (bp)	N (%)	Q20 (%)	Q30 (%)
HJY_0_1	42848806	6470169706	6086036368	0.001487	98	94.06
HJY_0_2	43727392	6602836192	6194160247	0.001479	97.92	93.81
HJY_0_3	45239370	6831144870	6423442764	0.001496	97.99	94.03
HJY_12_1	44950060	6787459060	6322060291	0.004169	97.56	93.14
HJY_12_2	42984276	6490625676	6048780489	0.004229	97.59	93.19
HJY_12_3	42842392	6469201192	6026793781	0.004200	97.56	93.16
HJY_24_1	47643042	7194099342	6703416910	0.004148	97.58	93.17
HJY_24_2	46256212	6984688012	6517304274	0.004149	97.63	93.3
HJY_24_3	42428030	6406632530	6009931474	0.004180	97.83	93.8
HJY_72_1	42664456	6442332856	6010849398	0.004126	97.63	93.3
HJY_72_2	44388036	6702593436	6233114606	0.004141	97.48	92.99
HJY_72_3	40957402	6184567702	5768319962	0.004131	97.62	93.26

Note: HJY_0_1, HJY_0_2 and HJY_0_3 indicated the three biological repeats in the leaves of ‘Huangjinya’ grown under natural light condition; HJY_12_x, HJY_24_x and HJY_72_x indicated the three biological repeats in the leaves of ‘Huangjinya’ shaded for 12h, 24h and 72h, respectively. Q30 (bp) referred the total number of bases with base recognition accuracy above 99.9 %; N (%) referred the percentage of fuzzy bases; Q20(%) referred the percentage of bases with base recognition accuracy above 99%; Q30(%) referred the percentage of bases with base recognition accuracy above 99.9%.

Table S4. Regional distribution of the clean reads mapping to the reference genome

Sample	Clean Reads	Map Events	Mapped to Gene	Mapped to InterGene	Mapped to Exon
HJY_0_1	40069978	30039334	25871564 (86.13%)	4167770 (13.87%)	24267301 (93.80%)
HJY_0_2	40939506	30520393	26361073 (86.37%)	4159320 (13.63%)	24762253 (93.93%)
HJY_0_3	42176878	31482644	26650332 (84.65%)	4832312 (15.35%)	24746138 (92.85%)
HJY_12_1	42118360	30936884	27131650 (87.70%)	3805234 (12.30%)	25698416 (94.72%)
HJY_12_2	40241200	29474987	25776455 (87.45%)	3698532 (12.55%)	24336254 (94.41%)
HJY_12_3	40129790	29444634	25651533 (87.12%)	3793101 (12.88%)	24172687 (94.23%)
HJY_24_1	44649514	32809511	28589454 (87.14%)	4220057 (12.86%)	26532782 (92.81%)
HJY_24_2	43373118	31930811	28010483 (87.72%)	3920328 (12.28%)	26180374 (93.47%)
HJY_24_3	39738814	29235707	25496246 (87.21%)	3739461 (12.79%)	23633836 (92.70%)
HJY_72_1	40008700	29409274	25801309 (87.73%)	3607965 (12.27%)	24174171 (93.69%)
HJY_72_2	41611276	30462189	26449730 (86.83%)	4012459 (13.17%)	24436414 (92.39%)
HJY_72_3	38298892	28099628	24600537 (87.55%)	3499091 (12.45%)	23029986 (93.62%)

Note: HJY_0_1, HJY_0_2 and HJY_0_3 indicated the three biological repeats in the leaves of ‘Huangjinya’ grown under natural light condition; HJY_12_x, HJY_24_x and HJY_72_x indicated the three biological repeats in the leaves of ‘Huangjinya’ shaded for 12h, 24h and 72h, respectively.

Table S5. Key differentially expressed genes involved in pathways of chlorophyll synthesis and degradation, LHCII subunit translocation and degradation as well as plastid retrograde signaling screened out from transcriptome analysis.

Gene ID	HJY0	HJY12	HJY24	HJY72	Function
GSAAT-CSS0005977	34.34	60.43	48.22	55.00	Chlorophyll synthesis
GluTR-CSS0041947	26.34	114.31	121.61	180.27	Chlorophyll synthesis
CPO-CSS0003887	58.86	96.56	67.42	70.14	Chlorophyll synthesis
UROS-CSS0036187	25.00	52.45	50.15	48.71	Chlorophyll synthesis
UROD-CSS0044621	95.41	156.89	132.40	130.03	Chlorophyll synthesis
CHLM-CSS0004907	76.26	165.32	104.82	87.90	Chlorophyll synthesis
PPOX-CSS0007697	23.56	47.11	41.17	46.31	Chlorophyll synthesis
POR-CSS0033593	23.01	393.82	359.43	440.45	Chlorophyll synthesis
CHL27-CSS0045826	18.81	48.24	52.12	79.76	Chlorophyll synthesis
CBR-CSS0001462	26.29	67.08	69.66	80.66	Chlorophyll synthesis
FC-CSS0012423	73.77	38.47	38.25	40.94	Chlorophyll synthesis
HY1-CSS0000206	20.75	9.75	10.20	9.12	Chlorophyll synthesis
PAO-CSS0024763	23.80	14.06	15.77	13.77	Chlorophyll breakdown
RCCR-CSS0031276	81.52	31.50	23.32	27.88	Chlorophyll breakdown
PSBP1-CSS0016981	54.37	546.16	523.31	610.03	LHCB translocation
GLK1-CSS0001889	6.19	3.33	1.94	5.92	LHCB translocation
TIC55-CSS0018677	4.01	1.84	1.48	1.17	LHCB translocation
TOC64-CSS0011043	6.97	1.03	1.90	2.52	LHCB translocation
TOC75-CSS0026296	76.95	38.89	31.83	34.29	LHCB translocation
TOC 159-CSS0025255	144.98	79.42	56.74	61.25	LHCB translocation
Hsp70-CSS0018653	506.08	17.30	32.17	46.15	LHCB translocation
Hsp90-CSS0018705	555.96	206.81	180.78	216.40	LHCB translocation
cpn60-CSS0018215	0.90	6.29	3.48	3.42	LHCB translocation
cpn60-CSS0029920	1.24	6.36	6.07	10.59	LHCB translocation
14-3-3 protein-CSS0027025	38.50	207.98	251.18	183.51	LHCB translocation
cpSRP54-CSS0014332	120.70	85.17	59.48	64.81	LHCB translocation
cpSRP54-CSS0046572	6.58	5.26	8.69	6.99	LHCB translocation
cpFtsY-CSS0006849	61.31	36.42	29.00	32.23	LHCB translocation
LTD-CSS0021065	133.84	137.46	101.23	110.64	LHCB translocation
ALB4-CSS0027798	129.62	63.00	52.01	50.48	LHCB translocation
Atg8-CSS0028099	13.56	35.42	34.23	26.77	LHCB degradation
Atg18-CSS0041332	2.50	10.50	8.84	9.60	LHCB degradation
Clp-CSS0009500	0.50	0.81	0.71	0.86	LHCB degradation
ClpPR4-CSS0029485	22.23	46.86	44.23	38.62	LHCB degradation
FtsH6-CSS0025086	0.20	3.80	8.48	10.42	LHCB degradation
FtsH10-CSS0008560	57.95	18.39	20.18	21.22	LHCB degradation
FtsH11-CSS0014825	2.20	2.46	1.53	1.95	LHCB degradation
SAGs-CSS0020038	67.89	7.37	4.91	5.24	LHCB degradation
GUN1-CSS0010117	52.75	25.76	18.34	18.00	Plastid retrograde signaling pathway
GUN1-CSS0036979	86.05	86.17	58.63	71.23	Plastid retrograde signaling pathway

PTM-CSS0018354	18.52	10.24	10.64	10.20	Plastid retrograde signaling pathway
ABI4-CSS0018330	40.57	7.84	6.23	6.92	Plastid retrograde signaling pathway
ABI4-CSS0005165	24.52	3.83	4.49	4.90	Plastid retrograde signaling pathway
EX1-CSS0042248	72.73	44.17	44.23	45.80	Plastid retrograde signaling pathway
EX1-CSS0042898	322.17	204.79	182.41	172.69	Plastid retrograde signaling pathway
cpAPX-CSS0040488	7.04	16.18	13.44	14.02	Plastid retrograde signaling pathway
FC2-CSS0006733	6.26	11.76	9.18	9.67	Plastid retrograde signaling pathway
GLK2-CSS0003354	0.68	1.20	3.21	2.91	Plastid retrograde signaling pathway
APX-CSS0034174	343.43	282.84	474.45	625.90	Plastid retrograde signaling pathway

Note: Data in table were the average values of the FPKM obtained from three biological repeats. HJY0 represented the leaves of 'HJY' grown under natural light condition, HJY12-72 represented the leaves of 'HJY' shaded for 12h, 24h and 72h, respectively.

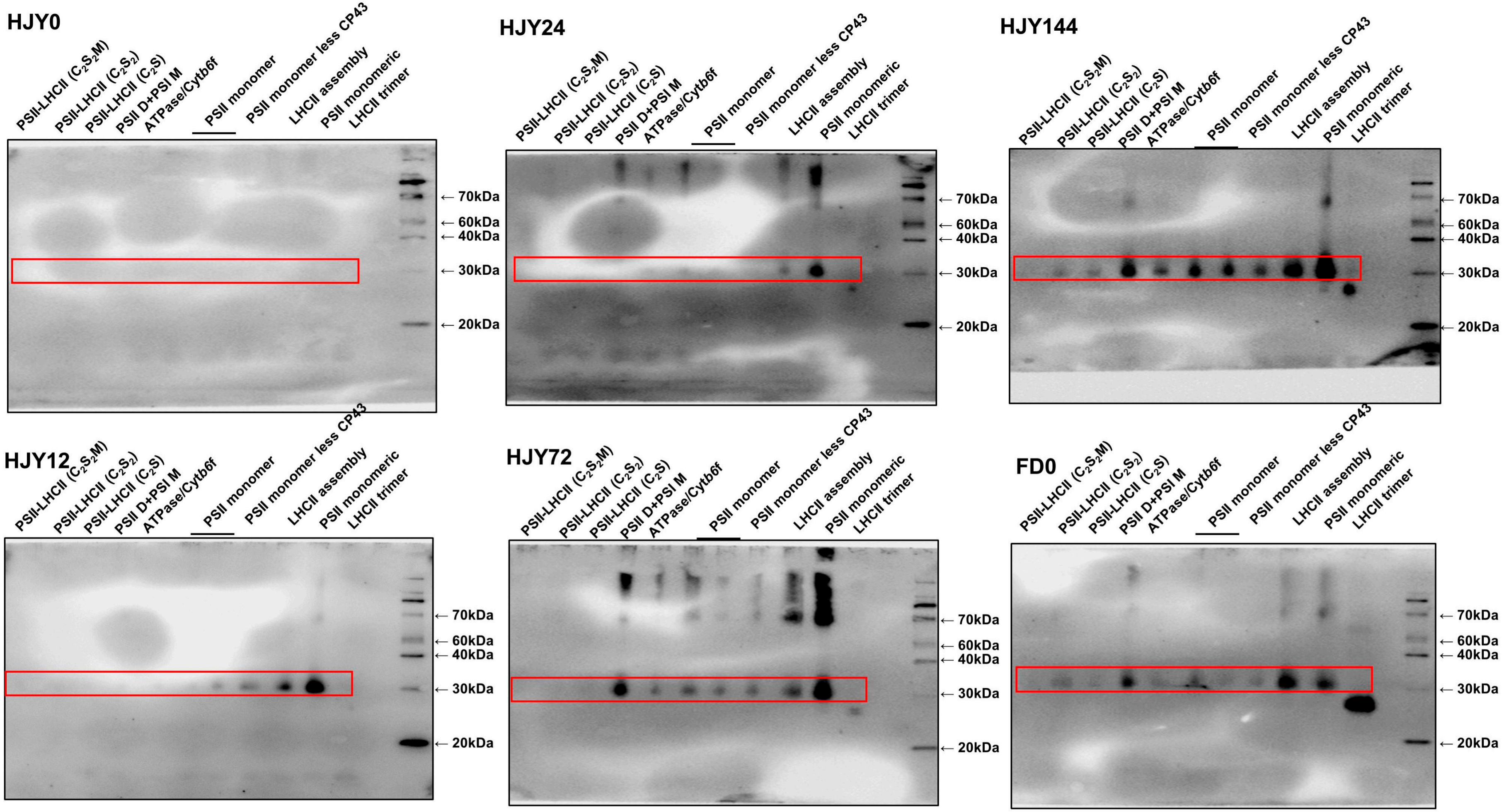

Figure S1. Original images for blots of Figure 6. In details, images exhibited D2 signals detected from various pigment-protein complexes in the leaves of 'HJY' during shading treatment. HJY0-HJY144 indicated the leaves of 'HJY' shaded for 0-144 h. FD0 indicated the leaves of 'FD' under natural light condition.

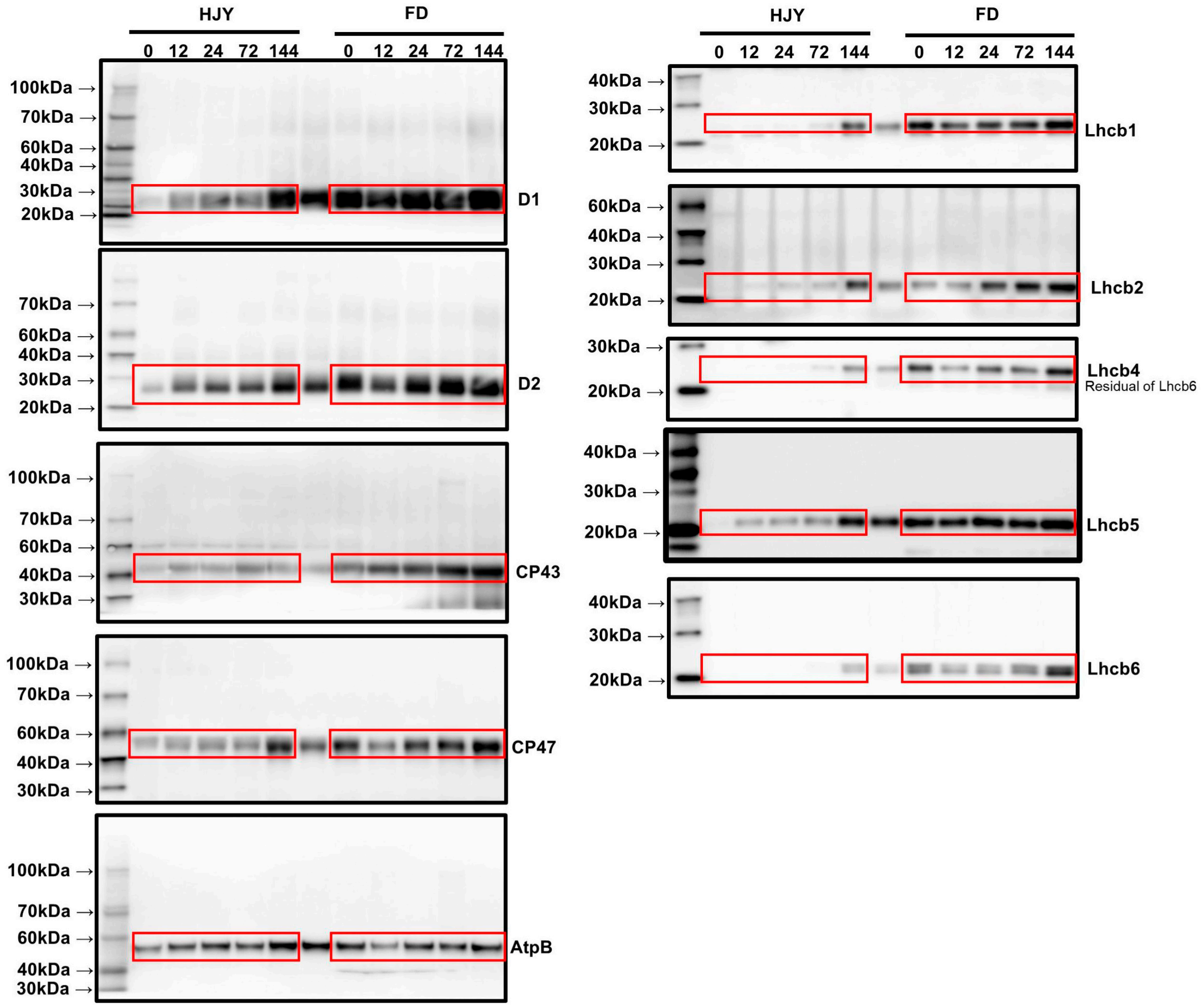

Figure S2. Original images for blots of Figure 7A. In details, images exhibited the changes in the PSII core complex and LHCII subunits during short-term shading treatment. HJY0-HJY144 and FD0-FD144 indicated the leaves of ‘HJY’ and ‘FD’ shaded for 0-144h.

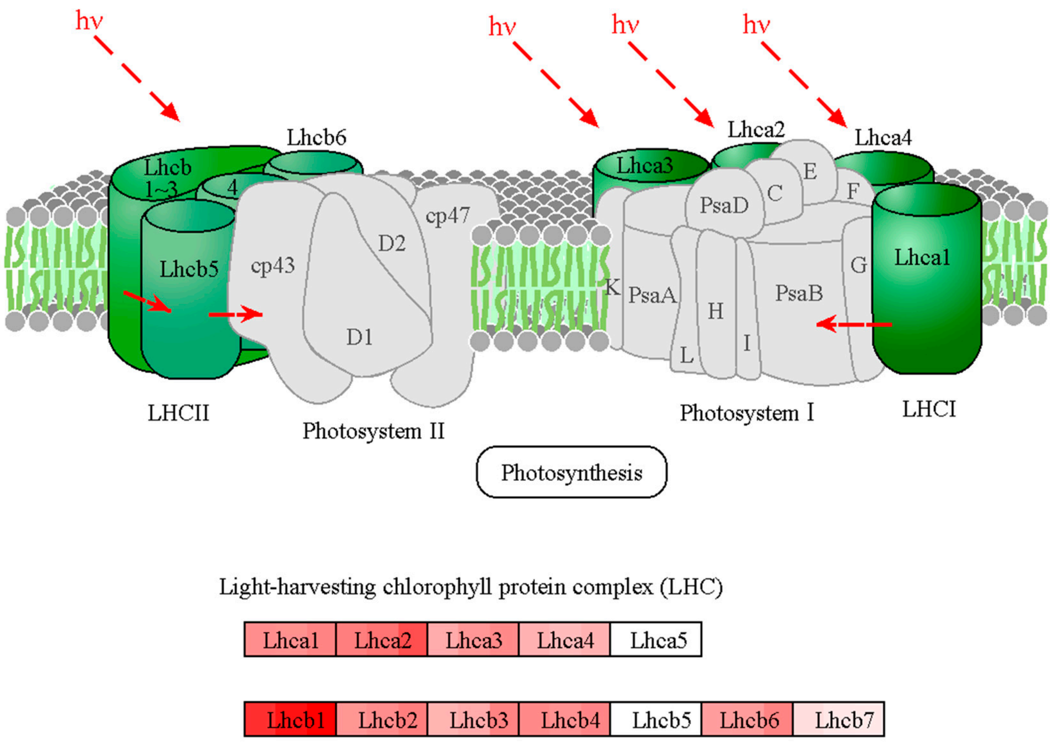

Figure S3. DEGs in photosynthesis-antenna protein pathway (Ko 00196). Red and blue blocks represented up- and down- regulations, respectively; the color intensity indicated the expression change degree of the DEGs. Each gene box was divided into three parts from left to right, representing the differential expression degree of the DEGs in comparison of HJY12 vs HJY0, HJY24 vs HJY0, and HJY72 vs HJY0.

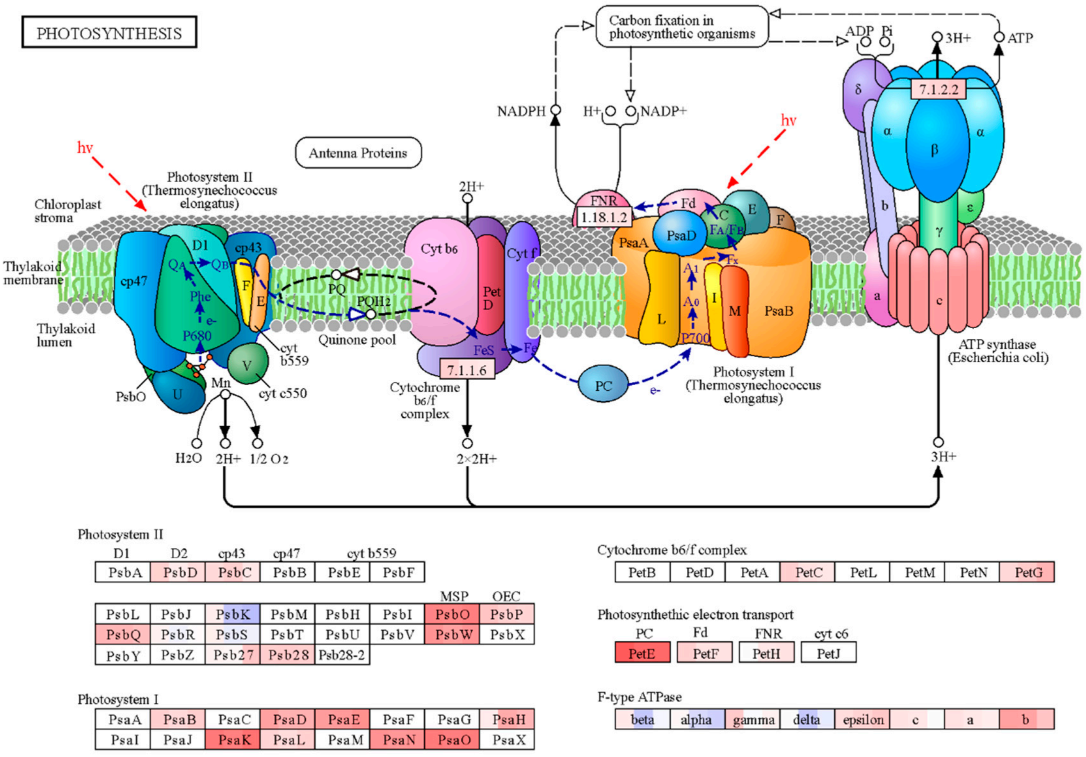

Figure S4. DEGs in photosynthesis pathway (Ko00195). Red and blue blocks represented up- and down-regulations, respectively; the color intensity indicated the expression change degree of the DEGs. Each gene box was divided into three parts from left to right, representing the differential expression degree of the DEGs in comparison of HJY12 vs HJY0, HJY24 vs HJY0, and HJY72 vs HJY0.
